# Supplementary material for: A modular chromosomally integrated toolkit for ectopic gene expression in Vibrio cholerae
Source: Sci Rep. 2020 Sep 21;10:15398. doi: 10.1038/s41598-020-72387-8 (PMC7505983; doi:10.1038/s41598-020-72387-8)
Supplement: Supplementary file 1 — Supplementary information [file 41598_2020_72387_MOESM1_ESM.pdf]

Supporting Information for

A modular chromosomally integrated toolkit for ectopic gene expression in *Vibrio cholerae*

Triana N. Dalia[1], Jennifer L. Chlebek[1], and Ankur B. Dalia[1,*]

[1]Department of Biology, Indiana University, Bloomington, IN 47405

*Author for correspondence: ankdalia@indiana.edu

This PDF file includes:

Fig. S1
Tables S1-S2

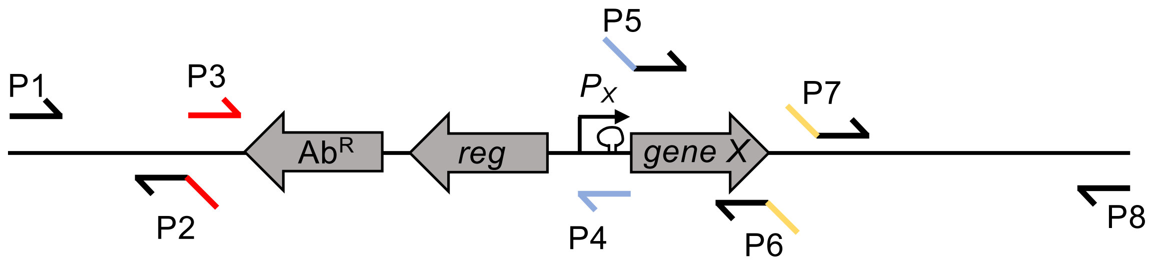

P1: BBC832 - GCTTTTTGCTACAACGACCG
P2: BBC828 - caccatacccacgccgaaacaaCAGTGATGTAGCGAATCGGAC
P3: BBC243 - TTGTTTCGGCGTGGGTATGGTG
P4-1 (P_{tac}-riboswitch or P_{BAD}-riboswitch): BBC2137 - CTTGTTGTTACCTCCTTAGCAGG
P5-1 (P_{tac}-riboswitch or P_{BAD}-riboswitch): cctgctaaggaggtaacaacaagATGXXXXXX
P4-2 (P_{tac} or P_{BAD}): BBC203 - CCCGGGATCCTGTGTGAAATTG
P5-2 (P_{tac} or P_{BAD}): caatttcacacaggatcccggg**AGGAGGT**NNNNNNATGXXXXXX
P6: tgtaggctggagctgcttcTTAYYYYYY
P7: BBC830 - gaagcagctccagcctacaGTTGAGTTGGATGCAGCACC
P8: BBC834 - CACAATTTCTCGCTTAAAATGTCC

Fig. S1 - *Details on how to assemble the ectopic expression constructs and swap out genes of interest.* A generic diagram of the ectopic expression construct is shown with the location of different primers needed to initially construct each expression construct (P1, P2, P3, P6, P7, and P8). As well as the primers to insert novel genes of interest into established expression constructs (P1, P4, P5, P6, P7, and P8). Ab^R = antibiotic resistance cassette, reg = regulatory gene (*lacIq* or *araC*), P_X = the promoter (P_{tac}, P_{BAD}, P_{tac}-riboswitch, or P_{BAD}-riboswitch), and gene X = the gene of interest.

Establishing ectopic expression constructs at new genomic locations: The UP arm of homology is generated with P1 and P2, while the DOWN arm of homology is amplified with P7 and P8. In this example, these primers amplify homology arms to integrate the ectopic expression constructs into *V. cholerae* ChII. P2 and P7 contain tails that overlap any of the 4 ectopic expression constructs. To move intact expression constructs to new locations in the genome, they can be amplified with P3 and P6. This can serve as a MIDDLE arm to stitch to the UP and DOWN arms of homology.

To insert new genes of interest:

Once an ectopic expression construct has been introduced to a genetic locus, the gene of interest can be swapped out.

-To amplify a gene of interest to place within P_{tac}-riboswitch or P_{BAD}-riboswitch the forward P5-1 primer must have a 5' overlap region as indicated. Immediately after this overlap should be the start codon for the gene of interest (highlighted in green) + additional sequence to serve as a primer for the gene of interest (denoted by 'XXXXXX'). P5-1 overlaps with P4-1 (blue highlighted sequence), which sits within the theophylline-dependent riboswitch. Thus, the same overlap can be used to make either P_{tac}-riboswitch or P_{BAD}-riboswitch constructs. The reverse P6 primer for the gene of interest must have the tail indicated, which should be immediately followed by the stop codon of the gene (highlighted in purple – TAA stop codon in this example) + additional sequence to serve as a primer for the gene of interest (denoted by 'YYYYYY').
-To amplify a gene of interest to place within P_{tac} or P_{BAD} the forward P5-2 primer must have the 5' overlap region indicated. Immediately after this overlap should be the desired ribosome binding site (indicated in bold and underline – the 'optimal' RBS AGGAGGT is used in this example), which should be followed by a 6 bp spacer (this spacing between the ribosome binding site and the start codon is essential for optimal translation and can be derived from the native gene) + the start codon (highlighted in green) + additional sequence to serve as a primer for the gene of interest (denoted by 'XXXXXX'). P5-2 overlaps with P4-2 (blue highlighted sequence), which sits downstream of the two promoter elements. Thus, the same overlap can be used to make either P_{tac} or P_{BAD} constructs. The reverse P6 primer for the gene of interest can be made exactly as described above.

All amplified genes of interest serve as MIDDLE arms in SOE reactions with an UP arm amplified with P1 and P4, and a DOWN arm amplified with P7 and P8.

Table S1 – *Strains used in this study*

Name	Relevant figure(s)	Full genotype	Reference / Strain#
parent	Fig. 2E	E7946 *lacZ*::FRT-Spec^R-FRT	This study / SAD035
P_{tac}-*gfp*	Fig. 2A, E; Fig. 3	E7946 *lacZ*::FRT-Spec^R-FRT, ΔVCA0692::P_{tac}-*gfp* Carb^R	This study / TND2290 (SAD2780)
P_{BAD}-*gfp*	Fig. 2B, E; Fig. 3	E7946 *lacZ*::FRT-Spec^R-FRT, ΔVCA0692::P_{BAD}-*gfp* Carb^R	This study / TND2291 (SAD2781)
P_{tac}-riboswitch-*gfp*	Fig. 2C, E; Fig. 3	E7946 *lacZ*::FRT-Spec^R-FRT, ΔVCA0692::P_{tac}-riboswitch-*gfp* Carb^R	This study / TND2293 (SAD2782)
P_{BAD}-riboswitch-*gfp*	Fig. 2D, E; Fig. 3	E7946 *lacZ*::FRT-Spec^R-FRT, ΔVCA0692::P_{BAD}-riboswitch-*gfp* Carb^R	This study / TND2292 (SAD2783)
wildtype	Fig. 4A	E7946	(25) / SAD031
P_{BAD}-*Flp*	Fig. 4A-B	E7946 *lacZ*::FRT-Spec^R-FRT, ΔVCA0692::P_{BAD}-*Flp* Carb^R	This study / TND2083 (SAD2784)
P_{tac}-riboswitch-*Flp*	Fig. 4B	E7946 *lacZ*::FRT-Spec^R-FRT, ΔVCA0692::P_{tac}-riboswitch-*Flp* Carb^R	This study / TND2289 (SAD2785)
P_{BAD}-riboswitch-*Flp*	Fig. 4B	E7946 *lacZ*::FRT-Spec^R-FRT, ΔVCA0692::P_{BAD}-riboswitch-*Flp* Carb^R	This study / TND2086 (SAD2786)
Δ*pilB*	Fig. 5A	E7946 Sm^R, P_{const}-tfoX, ΔluxO, *pilA* S67C, *lacZ*::FRT-Kan^R-FRT, ΔVC1807::CmR, Δ*pilB*	This study / TND2373 (SAD2787)
P_{tac}-*pilB* Δ*pilB*	Fig. 5A	E7946 Sm^R, P_{const}-tfoX, ΔluxO, *pilA* S67C, Δ*lacZ*::P_{tac}-*pilB* Spec^R, ΔVC1807::CmR, Δ*pilB*	This study / JLC769 (SAD2788)
P_{BAD}-*pilB* Δ*pilB*	Fig. 5A	E7946 Sm^R, P_{const}-tfoX, ΔluxO, *pilA* S67C, *lacZ*::FRT-Kan^R-FRT, ΔVC1807::CmR, Δ*pilB*, ΔVCA0692::P_{BAD}-*pilB* Carb^R	This study / TND2403 (SAD2789)
P_{tac}-riboswitch-*pilB* Δ*pilB*	Fig. 5A	E7946 Sm^R, P_{const}-tfoX, ΔluxO, *pilA* S67C, *lacZ*::FRT-Kan^R-FRT, ΔVC1807::CmR, Δ*pilB*, ΔVCA0692::P_{tac}-riboswitch-*pilB* Carb^R	This study / TND2402 (SAD2790)
P_{BAD}-riboswitch-*pilB* Δ*pilB*	Fig. 5A	E7946 Sm^R, P_{const}-tfoX, ΔluxO, *pilA* S67C, *lacZ*::FRT-Kan^R-FRT, ΔVC1807::CmR, Δ*pilB*, ΔVCA0692::P_{BAD}-riboswitch-*pilB* Carb^R	This study / TND2378 (SAD2791)
P_{tac}-*pilB* Δ*pilB* Δ*pilT*	Fig. 5B	E7946 Sm^R, P_{const}-tfoX, ΔluxO, *pilA* S67C, Δ*lacZ*::P_{tac}-*pilB* Spec^R, ΔVC1807::CmR, Δ*pilB*, Δ*pilT*::Zeo^R	This study / TND2498
P_{BAD}-*pilB* Δ*pilB* Δ*pilT*	Fig. 5B	E7946 Sm^R, P_{const}-tfoX, ΔluxO, *pilA* S67C, *lacZ*::FRT-Kan^R-FRT, ΔVC1807::CmR, Δ*pilB*, ΔVCA0692::P_{BAD}-*pilB* Carb^R, Δ*pilT*::Zeo^R	This study / TND2501

P_{tac}-riboswitch-*pilB* Δ*pilB* Δ*pilT*	Fig. 5B	E7946 Sm^R, P_{const}-tfoX, ΔluxO, *pilA* S67C, *lacZ*::FRT-Kan^R-FRT, ΔVC1807::CmR, Δ*pilB*, ΔVCA0692::P_{tac}-riboswitch-*pilB* $Carb^R$, Δ*pilT*::Zeo^R	This study / TND2500
P_{BAD}-riboswitch-*pilB* Δ*pilB* Δ*pilT*	Fig. 5B	E7946 Sm^R, P_{const}-tfoX, ΔluxO, *pilA* S67C, *lacZ*::FRT-Kan^R-FRT, ΔVC1807::CmR, Δ*pilB*, ΔVCA0692::P_{BAD}-riboswitch-*pilB* $Carb^R$, Δ*pilT*::Zeo^R	This study / TND2499

Table S2 – *Primers used in this study*

Primer Name	Primer Sequence (5'→3')	Description
BBC832	GCTTTTTGCTACAACGACCG	Replace VCA0692 w/ an ectopic expression construct UP ARM F1 (aka P1 in **Fig. S1**)
BBC828	caccatacccacgccgaaacaaCAGTGATGTAGCGAATCGGAC	Replace VCA0692 w/ an ectopic expression construct UP ARM R1 (aka P2 in **Fig. S1**)
BBC830	gaagcagctccagcctacaGTTGAGTTGGATGCAGCACC	Replace VCA0692 w/ an ectopic expression construct DOWN ARM F2 (aka P7 in **Fig. S1**)
BBC834	CACAATTTCTCGCTTAAAATGTCC	Replace VCA0692 w/ an ectopic expression construct DOWN ARM R2 (aka P8 in **Fig. S1**)
BBC243	TTGTTTCGGCGTGGGTATGGTG	Amplify any ectopic expression construct to move to a new genetic locus F (aka P3 in **Fig. S1**)
BBC203	CCCGGGATCCTGTGTGAAATTG	Primer to clone new ectopic genes into P_{tac} or P_{BAD} constructs R1 (aka P4-2 in **Fig. S1**)
BBC2137	CTTGTTGTTACCTCCTTAGCAGG	Primer to clone new ectopic genes into P_{tac}-riboswitch or P_{BAD}-riboswitch constructs R1 (aka P4-1 in **Fig. S1**)
BBC252	caatttcacacaggatcccgggAGGAGGTAACGTAATGCGTAAAGGAGAAGAAC	F Primer to amplify up *gfp* to clone into P_{tac} or P_{BAD} constructs (aka P5-2 in **Fig. S1**)
BBC2182	cctgctaaggaggtaacaacaagATGCGTAAAGGAGAAGAACTTTTCAC	F Primer to amplify up *gfp* to clone into P_{tac}-riboswitch or P_{BAD}-riboswitch (aka P5-1 in **Fig. S1**)
BBC254	tgtaggctggagctgcttcTTAGTTGTATAGTTCATCCATGCC	R Primer to amplify up *gfp* to clone into any ectopic expression construct (aka P6 in **Fig. S1**)
BBC2427	caatttcacacaggatcccgggAGGAGGTTTTTGTATGCCACAATTTGATATATTATG	F Primer to amplify up *Flp* to clone into P_{tac} or P_{BAD} constructs (aka P5-2 in **Fig. S1**)
BBC2455	cctgctaaggaggtaacaacaagATGCCACAATTTGATATATTATGTAAAAC	F Primer to amplify up *Flp* to clone into P_{tac}-riboswitch or P_{BAD}-riboswitch (aka P5-1 in **Fig. S1**)
BBC2428	tgtaggctggagctgcttcTTATATGCGTCTATTTATGTAGGATG	R Primer to amplify up *Flp* to clone into any ectopic expression construct (aka P6 in **Fig. S1**)
BBC1952	caatttcacacaggatcccgggAGGAGGTTAACTAATGCTCACCAACCTTGTTG	F Primer to amplify up *pilB* to clone into P_{tac} or P_{BAD} constructs (aka P5-2 in **Fig. S1**)
BBC2806	cctgctaaggaggtaacaacaagATGCTCACCAACCTTGTTGC	F Primer to amplify up *pilB* to clone into P_{tac}-riboswitch or P_{BAD}-riboswitch (aka P5-1 in **Fig. S1**)

BBC1953	tgtaggctggagctgcttcTTAAAAGTAGAGCACACGCTG	R Primer to amplify up *pilB* to clone into any ectopic expression construct (aka P6 in **Fig. S1**)
ABD253	GCGACCCCACCGATGGG	*lacZ*::FRT-Ab^R-FRT F1
ABD263	gtcgacggatccccggaatAACTGATCCAATTTTTCAGCGCATATTTTGG	*lacZ*::FRT-Ab^R-FRT R1
ABD262	gaagcagctccagcctacaTGCCGCAGGAAAACCGCCCCCTaATC	*lacZ*::FRT-Ab^R-FRT F2
ABD256	CCCAAATACGGCAACTTGGCG	*lacZ*::FRT-Ab^R-FRT R2
ABD123	ATTCCGGGGATCCGTCGAC	Amplify any Ab^R cassette F
ABD124	TGTAGGCTGGAGCTGCTTC	Amplify any Ab^R cassette R
